# Supplementary figures and images for: Glycan Masking of Epitopes in the NTD and RBD of the Spike Protein Elicits Broadly Neutralizing Antibodies Against SARS-CoV-2 Variants
Source: Front Immunol. 2021 Dec 2;12:795741. doi: 10.3389/fimmu.2021.795741 (PMC8674692; doi:10.3389/fimmu.2021.795741)

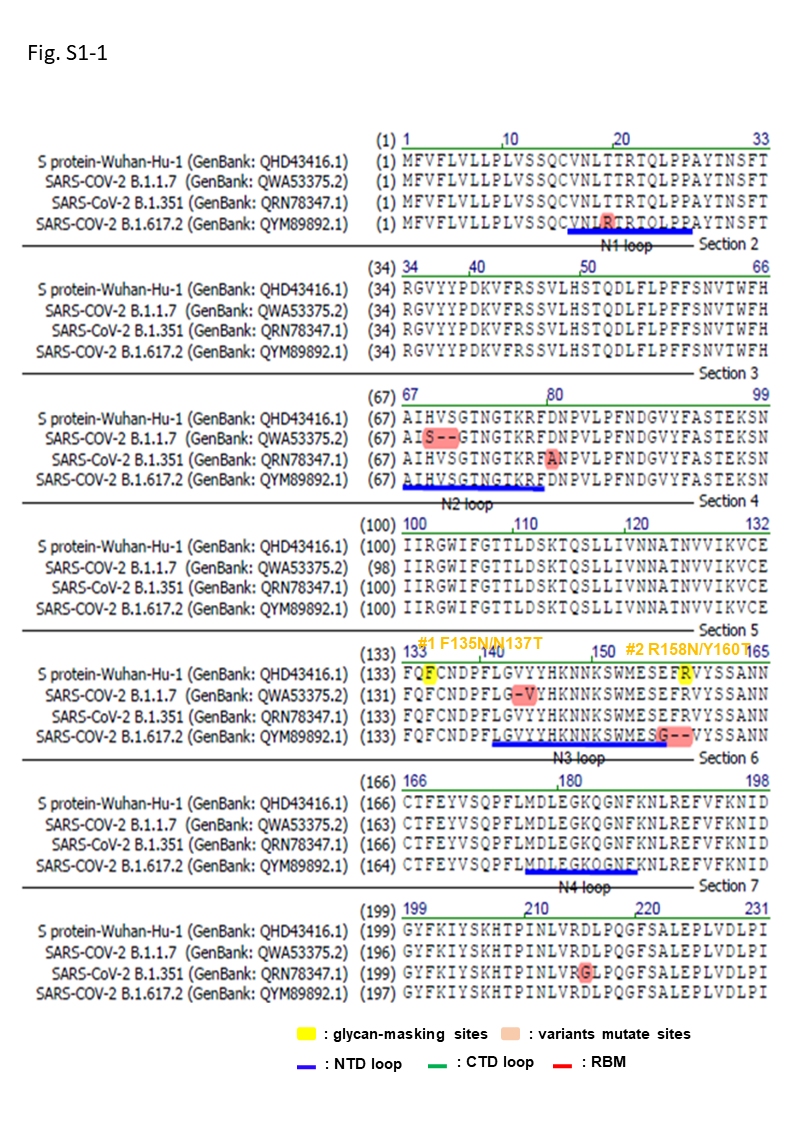

Supplement: Supplementary Figure 1 — Amino acid sequence alignment of SARS-CoV-2 Wuhan-Hu-1, Alpha (B.1.1.7), Beta (B.1.351), and Delta (B.1.617.2) strains. The NTD loops (blue), the CTD loops (green), and the receptor-binding motif (RBM, red) in the S protein are indicated. The glycan-masking sites (yellow) and variant mutation sites (pink) are marked. The CTD loops are the C-terminal domain loops of S1 subunit. [file Image_1.tif]

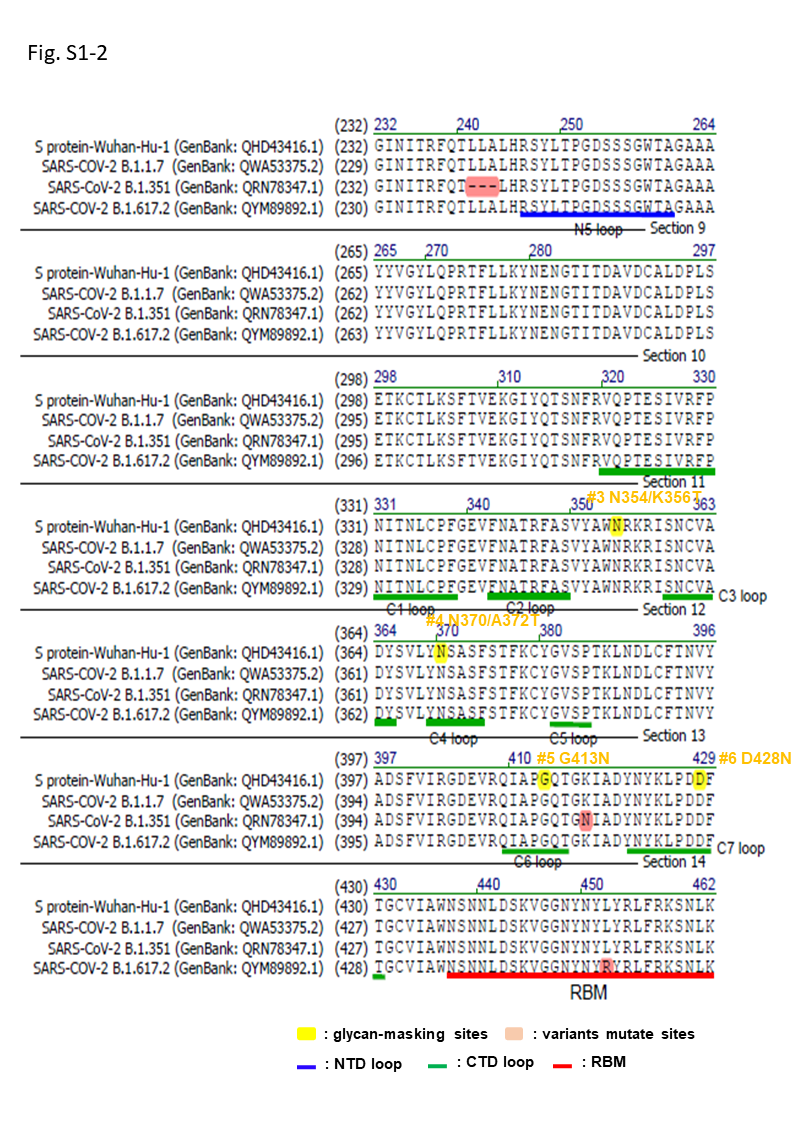

Supplement: Supplementary file 2 [file Image_2.tif]

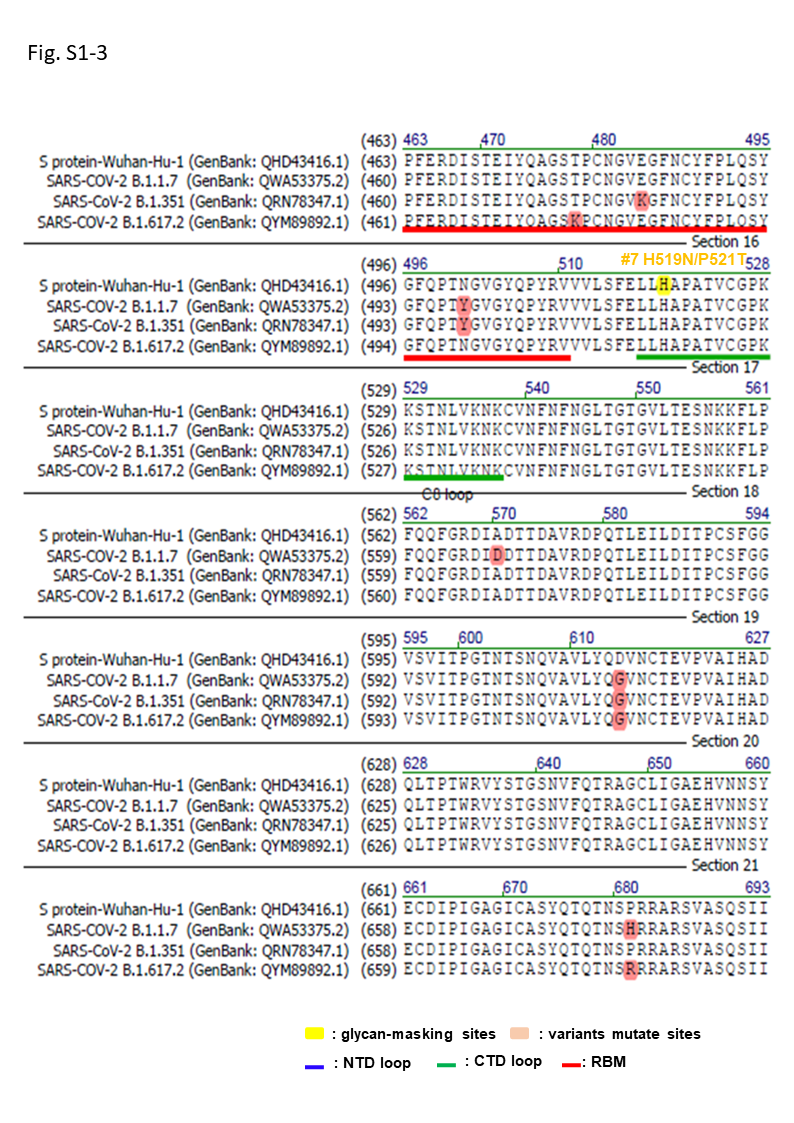

Supplement: Supplementary file 3 [file Image_3.tif]
